# The Ca^2+^-dependent protein kinase CPK3 is required for MAPK-independent salt-stress acclimation in Arabidopsis

**DOI:** 10.1111/j.1365-313X.2010.04257.x

**Published:** 2010-06-15

**Authors:** Norbert Mehlmer, Bernhard Wurzinger, Simon Stael, Daniela Hofmann-Rodrigues, Edina Csaszar, Barbara Pfister, Roman Bayer, Markus Teige

**Affiliations:** Department of Biochemistry and Cell Biology, Max F. Perutz Laboratories, University of ViennaDr. Bohrgasse 9/5, A-1030 Vienna, Austria

**Keywords:** Ca^2+^-dependent protein kinase, salt stress adaptation, MAP kinase, crosstalk, *N*-myristoylation, protein phosphorylation

## Abstract

Plants use different signalling pathways to respond to external stimuli. Intracellular signalling via calcium-dependent protein kinases (CDPKs) or mitogen-activated protein kinases (MAPKs) present two major pathways that are widely used to react to a changing environment. Both CDPK and MAPK pathways are known to be involved in the signalling of abiotic and biotic stresses in animal, yeast and plant cells. Here, we show the essential function of the CDPK CPK3 (At4g23650) for salt stress acclimation in *Arabidopsis thaliana*, and test crosstalk between CPK3 and the major salt-stress activated MAPKs MPK4 and MPK6 in the salt stress response. CPK3 kinase activity was induced by salt and other stresses after transient overexpression in Arabidopsis protoplasts, but endogenous CPK3 appeared to be constitutively active in roots and leaves in a strictly Ca^2+^-dependent manner. *cpk3* mutants show a salt-sensitive phenotype comparable with mutants in MAPK pathways. In contrast to animal cells, where crosstalk between Ca^2+^ and MAPK signalling is well established, CPK3 seems to act independently of those pathways. Salt-induced transcriptional induction of known salt stress-regulated and MAPK-dependent marker genes was not altered, whereas post-translational protein phosphorylation patterns from roots of wild type and *cpk3* plants revealed clear differences. A significant portion of CPK3 was found to be associated with the plasma membrane and the vacuole, both depending on its *N*-terminal myristoylation. An initial proteomic study led to the identification of 28 potential CPK3 targets, predominantly membrane-associated proteins.

## Introduction

Plants as sessile organisms must respond to changes in environmental conditions, such as different forms of stress or different light intensities. Many extracellular signals elicit changes in the cellular Ca^2+^ concentrations in plants (Cheng *et al.*, 2002; Sanders *et al.*, 2002; Ludwig *et al.*, 2004). The decoding of these calcium signals is performed by protein kinases, such as the calcium-dependent protein kinases (CDPKs), that mediate cellular responses either directly by changing enzymatic activities via protein phosphorylation or indirectly by changing gene expression patterns (Sathyanarayanan and Poovaiah, 2004). Additional signal transduction pathways use evolutionary highly conserved mitogen-activated protein kinase (MAPK) cascades to transmit extracellular signals towards altered gene expression patterns, resulting in cellular adaptation (Hamel *et al.*, 2006; Colcombet and Hirt, 2008). Whereas crosstalk between Ca^2+^-dependent signalling and MAPK pathways has been elaborately studied in animal systems (Agell *et al.*, 2002; Rozengurt, 2007), it has hardly been addressed in plant signal transduction.

Generally, these signalling pathways mediate changes in gene expression by modifiying the transcriptional machinery (Xiong *et al.*, 2002), ultimately leading to altered gene expression patterns. Accordingly, transcription factors have long been suspected to be important targets of those pathways. In addition, more and more evidence for direct modulation of enzymatic activities by protein phosphorylation has been reported in recent years. Stress-induced ethylene production is an example where different signalling pathways trigger ethylene synthesis by the activation of ACC synthase, the key enzyme for ethylene synthesis, at both levels: by transcriptional upregulation (Kim *et al.*, 2003; Teige *et al.*, 2004) or by direct protein phosphorylation (Liu and Zhang, 2004), thereby regulating protein stability and accumulation (Joo *et al.*, 2008). A putative CDPK phosphorylation site was also identified in ACC synthase (Hernandez Sebastia *et al.*, 2004), and ethylene was furthermore reported to mediate crosstalk between calcium dependent and MAPK signalling pathways (Ludwig *et al.*, 2005). Evidence for other targets of CDPKs, including different metabolic enzymes such as nitrate reductase (NR), sucrose synthase (SuSy) or sucrose phosphate synthase (SPS), has been summarized by Cheng *et al.* (2002), Harper *et al.* (2004) and Harper and Harmon (2005).

Salt stress constrains plant growth based on two effects: by causing osmotic stress, and by disturbing cellular ion homeostasis. Thus, the ability to maintain an optimal K^+^/Na^+^ ratio in the cytosol is one of the key features of plant salt tolerance (Tuteja, 2007; Munns and Tester, 2008; Leidi *et al.*, 2010). Accordingly, the regulation of numerous K^+^ uptake as well as K^+^ and Na^+^ transport systems is of major importance for adaptation to salt stress, and has been intensively studied in order to understand and improve salt tolerance in plants (Yamaguchi and Blumwald, 2005; Horie *et al.*, 2009). Plants apply a plethora of different systems to either exclude Na^+^ from the cell or to sequester it into the vacuole via Na^+^/H^+^ antiporters (Tuteja, 2007). Accordingly, the overexpression of the vacuolar Na^+^/H^+^ antiporter NHX1 (Apse *et al.*, 1999), or the plasma membrane Na^+^/H^+^ antiporter SOS1 (Shi *et al.*, 2003), conferred a remarkable salt tolerance to plants*.*

Elevated cytosolic Ca^2+^ levels are among the first cellular responses to extracellular salt stress (Knight *et al.*, 1997), and have been identified as a central regulator in numerous different stress signal transduction pathways (Xiong *et al.*, 2002; Zhu, 2002): for example, in the salt overly sensitive (SOS) pathway (Mahajan *et al.*, 2008). SOS3, a Ca^2+^ sensor, transduces the signal downstream by activating the protein kinase SOS2, which regulates the plasma membrane Na^+^/H^+^ antiporter SOS1, thus maintaining cellular ion homeostasis under salt stress conditions. The important role of Ca^2+^ signalling during the salt stress response is furthermore reflected by the findings that SOS2 also interacts with NHX1 (Qiu *et al.*, 2004), the vacuolar H^+^-ATPase (Batelli *et al.*, 2007) and the vacuolar H^+^/Ca^2+^ antiporter CAX1 (Cheng *et al.*, 2004), thus regulating cellular ion homeostasis in many different ways.

A recent analysis of *cpk3 cpk6* double knock-out plants has shown altered responses of vacuolar potassium channels in leaf guard cells in response to abscisic acid (ABA), suggesting that these two kinases regulate the activity of potassium channels by phosphorylation (Mori *et al.*, 2006). Interestingly, an increasing body of data on the potential regulation of membrane proteins by phosphorylation, including potential CDPK target motifs, appears in the current literature, pointing towards a much more complex network of Ca^2+^ signalling (Morel *et al.*, 2006; Marmagne *et al.*, 2007; Chang *et al.*, 2009).

In this work, we have analysed CPK3-mediated Ca^2+^-dependent signalling in the salt stress response in *Arabidopsis thaliana*. CPK3 had not previously been functionally characterized in detail, and accordingly its role in the salt stress response was absolutely unknown. The salt-sensitive phenotype of *cpk3* mutants could not be explained by the transcriptional induction of known salt-responsive genes, indicating that CPK3 acts primarily at the post-translational level and mediates the immediate stress response via the regulation of membrane-localized target proteins, whereas the MKK2–MPK4/6 pathway seems to be responsible for the transcriptional acclimation to salt stress. This model is supported by the identification of 28 potential CPK3 targets in an initial proteomic approach, which are predominantly membrane associated.

## Results

### CPK3 kinase activity is stress-induced in protoplasts but constitutive *in planta*

**Figure 1:**
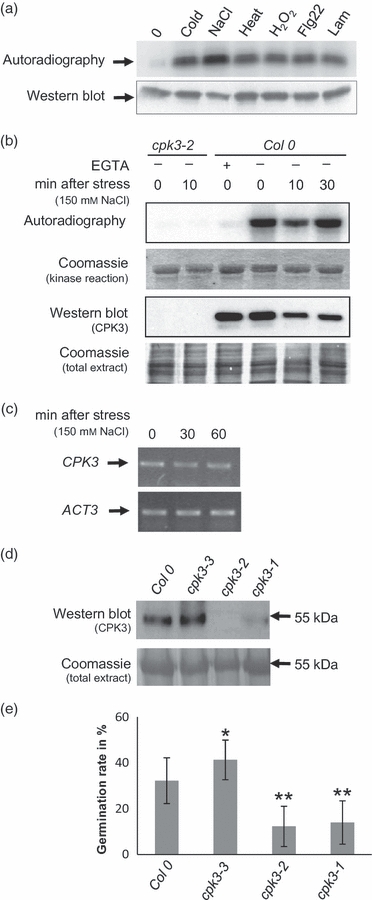
CPK3 kinase activities and salt-sensitive phenotype of *cpk3* mutants.(a) Activation of transiently expressed HA-epitope tagged CPK3 in response to different stresses in Arabidopsis protoplasts. Immunocomplex kinase assays were performed as described in Experimental procedures. The top panel shows the incorporation of ^32^P into the generic substrate Histone IIIS, and the bottom panel displays CPK3 protein levels: 0, mock treatment; cold, 4°C; NaCl, 150 mm; heat, 37°C; H_2_O_2_, 1 mm; flagellin, 15 nm; laminarin, 1 mm.(b) Endogenous CPK3 kinase activity in response to salt stress in roots of *cpk3-2* knock-out and Col-0 plants measured in immunocomplex kinase assays. Plants were treated with 150 mm NaCl for 0, 10 and 30 min. Immunocomplex kinase assays were performed in the absence or presence 200 μm EGTA.(c) *CPK3* transcript levels in response to salt stress.(d) CPK3 protein levels in Col-0 and three independent T-DNA insertion lines.(e) Germination rates of Col-0 and the T-DNA insertion lines on quarter-strength Hoagland + 150 mm NaCl. Error bars indicate SEM (*n* = 10). Statistically significant differences from Col-0 calculated by a two-tailed Student’s *t*-tests: *P ≤ 0.05; **P ≤ 0.001.

In order to attribute a biological function to CPK3, we analysed which conditions trigger its kinase activity. We first expressed haemagglutinin (HA) epitope-tagged CPK3 transiently in Arabidopsis protoplasts, and measured its kinase activity towards histone III-S as the substrate in immunocomplex kinase assays 15 min after the application of different stimuli. CPK3 kinase activity was found to be induced by almost all treatments performed, but clearly the strongest activation was detected after salt-stress treatment (Figure 1a). To verify these data *in planta*, we tested the salt-triggered activation of CPK3 in the roots and leaves of 6-week-old hydroponically grown plants and seedlings in immunocomplex kinase assays using a CPK3-specific antibody. This peptide antibody is directed against the C-terminal 15 amino acids of CPK3 and is highly specific, as indicated by the complete lack of detectable kinase activity in *cpk3* knock-out plants (*cpk3-2*; see Figure S1 for a detailed characterization of used lines). CPK3 kinase activity was completely abolished by the addition of 200 μm EGTA, but in contrast to the transiently overexpressed kinase in protoplasts, endogenous CPK3 appeared to be constitutively active in roots (Figure 1b), leaves and seedlings (Figure S2a,b) *in planta.* However, this apparent contradiction might be caused by technical limitations (see Discussion). In order to test whether *CPK3* is transcriptionally regulated in response to salt stress, we performed semi-quantitative RT-PCR using *CPK3*-specific primers and actin (*ACT3*; see Table S1 for primers) as the control (Figure 1c). Again, no significant difference in the expression level of *CPK3* could be detected in response to salt stress after 30 and 60 min, which is in line with *CPK3* expression data from microarrays (Kreps *et al.*, 2002).

### cpk3 plants display a salt-sensitive phenotype

To address whether CPK3 does play an essential role in the salt stress response of plants, we analysed different CPK3 knock-out and overexpressor lines for a phenotype under salt stress conditions. Three different T-DNA insertion lines for *CPK3* were obtained from the Salk collection (http://signal.salk.edu) (Alonso *et al.*, 2003). The position of their insertions was determined by PCR, and *CPK3* expression was measured at the mRNA and protein levels (Figures 1d and S1). The *cpk3-2* line was verified as a completely null mutant at the mRNA and protein level; the *cpk3-1* line showed remaining transcript, but almost no protein in the western blot. In contrast, the *cpk3-3* line, with insertion in the promoter region, had much higher protein levels compared with the corresponding wild type (Col-0) (Figure 1d). These lines were used to compare the germination rates of *cpk3* mutants and overexpressor lines with the corresponding wild type on agar plates containing 150 mm NaCl (Figure 1e). Without salt stress the germination rate of all lines was 100%, whereas on plates containing 150 mm NaCl a clear difference appeared. Germination of both *cpk3* mutant lines (*cpk3-2* and *cpk3-1*) was severely impaired compared with the wild type, whereas the germination rate of the *CPK3* overexpressing line (*cpk3-3*) was clearly increased. Moreover, the increase in the germination rate on salt corresponded with the level of *CPK3* expression, as revealed by the analysis of different *35S::CPK3-YFP* lines (Figure S3). In the *CPK3-1* line, representing a weak overexpressor according to the RT-PCR data, the salt tolerance was only slightly improved compared with the wild type, but the strong overexpressor line *CPK3-2* showed an improved germination rate under salt stress conditions. These differences proved to be statistically significant in both cases after analysis of data obtained from more than 100 seedlings per plate in 10 independent repetitions for each line tested. In summary, an essential role of CPK3 for adaptation to salt stress can be clearly concluded from these experiments.

### CPK3 is *N*-myristoylated, and localized to the nucleus and cellular membranes

**Figure 2:**
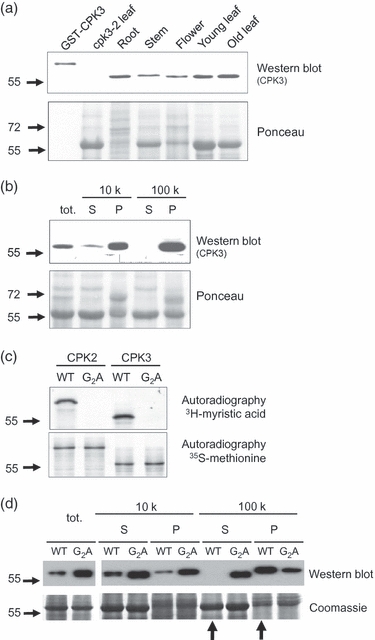
Localization and *N*-myristoylation of CPK3.(a) Tissue-specific expression of *CPK3* in plants: root, stem, flower, young (20 days post germination) and old (40 days post germination) leaves.(b) Endogenous CPK3 in subcellular fractionation from wild-type plants. Lanes from left to right: total cell extract, protein from the 10 000 ***g*** (10 k) supernatant (S) and pellet (P), as well as the 100 000 ***g*** (100 k) supernatant (S) and pellet (P).(c) *In vitro* myristoylation of CPK3 and CPK2 as a positive control. Wild-type (WT) and non-myristoylable G_2_A mutants of CPK2 and CPK3 were *in vitro* translated in the presence of either ^3^H-labelled myristic acid or ^35^S-labelled methionine, and incorporation of the label was scored by autoradiography.(d) Fractionation of YFP-tagged CPK3 (WT) and G_2_A mutants (G2A) from infiltrated tobacco leaves using a GFP antibody. S, supernatant; P, pellet; 10 k, centrifugation for 10 min at 10 000 ***g***; 100 k, centrifugation for 1 h at 100 000 ***g***.

Next, we addressed the question of tissue-specific and subcellular localization of CPK3. We compared endogenous CPK3 protein accumulation in different plant tissues and subcellular fractions of wild-type plants using the CPK3-specific antibody described above (Figure 2a). The antibody correctly recognized the recombinant protein, and no background was visible in the *cpk3-2* knock-out line. CPK3 protein could be detected in all tissues, which corresponds to transcript levels of published microarray data (Zimmermann *et al.*, 2004).

The further elucidation of the subcellular localization of CPK3 was particularly important because a chloroplast localization of CPK3 is predicted in several databases (i.e. MIPS or TAIR), and would be consistent with older published data on CPK3 (Hong *et al.*, 1996). In a more recent paper, a cytoplasmic and nuclear localization of CPK3 was shown in roots (Dammann *et al.*, 2003), but no data from leaves were shown in that work. We addressed CPK3 localization by two different approaches.

Subcellular fractionation experiments, detecting endogenous CPK3 by western blotting (Figure 2b), revealed that most of the protein had already accumulated in the first pellet obtained after 10 min of centrifugation at 10 000 ***g***. After centrifugation of the supernatant from the first separation for 1 h at 100 000 ***g***, almost no CPK3 remained detectable in the supernatant, emphasising its strong membrane association. The first pellet contained nuclei and chloroplasts, thus a more detailed analysis was required to determine the subcellular localization of CPK3. To this end, we studied the localization of CPK3-YFP fusion proteins in transiently transformed leaf epidermal cells, and found CPK3-YFP protein predominantly localized at cellular membranes and in the nucleus (Figure S4a), whereas free YFP was visible exclusively in the nucleus and in the cytosol, but not at membranes (Figure S4c). In some cases CPK3 seemed to co-localize with chloroplasts, but *in vitro* chloroplast import assays clearly showed that CPK3 could not be imported into chloroplasts (Figure S4d,e). These results were consistent with the subcellular fractionation of the endogenous protein, implying that the observed localization is not a result of overexpression artifacts.

As CPK3 harbours a potential myristoylation site in its N terminus, MGHRHSKSKS (Towler *et al.*, 1988; Qi *et al.*, 2000), we set out to test the possibility of *N*-myristoylation, and the functional consequences of this modification for localization. We performed an *in vitro* translation of CPK3 in wheatgerm extracts, and monitored the incorporation of either ^35^S-labelled methionine, or ^3^H-labelled myristic acid by autoradiography after SDS-PAGE (Figure 2c) to test whether CPK3 could be *N*-myristoylated, despite its negative prediction for this modification (Cheng *et al.*, 2002; Hrabak *et al.*, 2003). This approach had successfully been used before for CPK2 (Lu and Hrabak, 2002), and also revealed the *N*-myristoylation of CPK3 here. As negative controls, we used the non-myristoylable G_2_A mutant. As additional backup to prove the functional effect of *N*-myristoylation on the localization of CPK3, we performed biochemical fractionation experiments. Tobacco leaves were infiltrated with the wild-type and the G_2_A version of CPK3-YFP, and membrane association was detected by western blots using a GFP antibody (Figure 2d). No signal was detectable in the wild-type pellet of the 100 000 ***g*** supernatant, but a strong signal remained in the 100 000 ***g*** supernatant of the G_2_A mutant (indicated by the arrows in Figure 2d), clearly demonstrating that *N*-myristoylation is required for the membrane association of CPK3. To visualize the potential effects *in planta*, we transformed a construct in which the myristoylated amino acid glycine 2 had been substituted by an alanine (G_2_A), thus aborting the *N*-myristoylation of CPK3. As expected, the G_2_A mutant was no longer membrane associated, and displayed a strong accumulation in the nucleus and the cytosolic lobes of the epidermal cells (triangle in Figure S4b).

### *N*-myristoylation of CPK3 is required for its association with the vacuolar and plasma membrane

**Figure 3:**
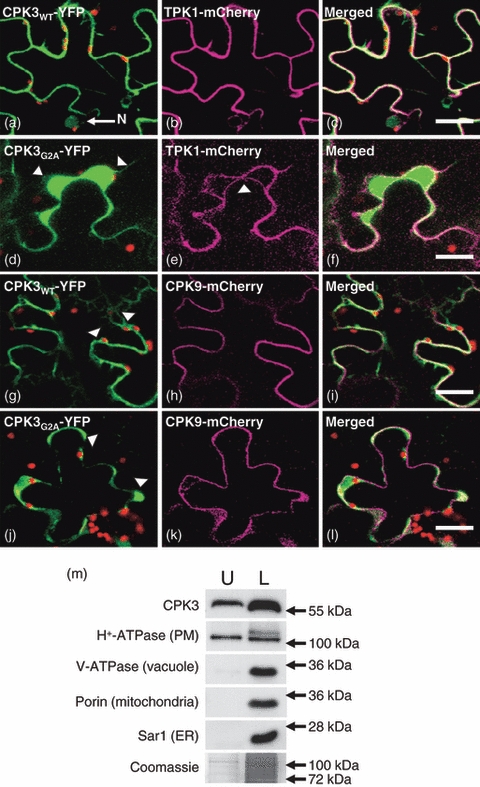
Co-localization of wild-type and non-myristoylated CPK3 with the vacuolar and the plasma membrane in tobacco leaf epidermal cells. The green channel shows the CPK3-YFP signal, the red channel displays chlorophyll autofluorescence and the magenta channel displays the mCherry fusion proteins. Co-localization can be deduced from a white signal in the merged images.(a–c) Co-localization of CPK3_WT_-YFP and TPK1-mCherry at vacuolar membranes. The nucleus (N) is marked by an arrow.(d–f) Co-expression of CPK3_G2A_-YFP and TPK1-mCherry.(g–i) Co-localization of CPK3_WT_-YFP and CPK9-mCherry at the plasma membrane.(j–l) Co-expression of CPK3_G2A_-YFP and CPK9-mCherry. Scale bars: 10 μm.(m) Co-partitioning of endogenous CPK3 with different membranes from Arabidopsis leaves in phase-partitioning experiments. The upper phase (U) contains purified plasma membrane; the lower phase (L) contains plasma, mitochondrial, vacuolar and endoplasmic reticulum membranes. Different membranes were detected via western blotting using the indicated markers.

In order to identify the specific membrane system to which CPK3 is localized *in vivo*, we co-expressed CPK3-YFP fusion proteins together with markers for the vacuolar membrane (TPK1; Latz *et al.*, 2007) and the plasma membrane (CPK9; Nelson *et al.*, 2006; Benetka *et al.*, 2008) as mCherry fusion proteins. We could clearly observe the co-localization of CPK3_WT_-YFP with the vacuolar two-pore potassium channel (TPK1) at the vacuolar membrane (Figure 3a–c). In contrast, the non-myristoylatable CPK3_G2A_-YFP accumulated in cytoplasmic lobes (triangles in Figure 3d), whereas TPK1-mCherry was restricted to the tonoplast (triangle in Figure 3e). If visible, the position of the nucleus was deduced from the bright-field images (Figure S5). The effect of the *N*-myristoylation of CPK3 is clearly visible by comparing the merged images of the wild-type protein with TPK1 (Figure 3c) and the G_2_A mutant (Figure 3f). Similarly, we could observe the co-localization of CPK3_WT_-YFP and the plasma membrane localized CPK9-mCherry (Figure 3g–i). Here, CPK3 was also visible in cytoplasmic regions, for example in the areas surrounding the chloroplasts (triangles in Figure 3g), whereas CPK9 was restricted to the plasma membrane (Figure 3h). Again, the non-myristoylatable CPK3_G2A_-YFP appeared in cytoplasmic lobes (triangles in Figure 3j), whereas co-expressed CPK9-mCherry labelled the plasma membrane (Figure 3k), and the visible co-localization is reduced (Figure 3l) compared with the wild-type CPK3 (Figure 3i). In order to backup these results with an independent biochemical approach, we isolated plasma membranes from a microsomal membrane fraction by two-phase partitioning from Arabidopsis leaves to detect endogenous CPK3. This method has also been used for CPK2 (Lu and Hrabak, 2002), and enriches the plasma membrane (PM) in the upper phase and other cellular membranes in the lower phase. Both phases were analysed by western blotting to detect CPK3 and markers for the plasma membrane (H^+^-ATPase), vacuolar membrane (V-ATPase), mitochondrial membranes (Porin) and the endoplasmic reticulum (ER) (Sar1) (Figure 3m). Consistent with the previous results obtained by microscopy, CPK3 did partition with the PM in the upper phase (U), and could also be detected in the lower phase (L), together with the other membrane fractions.

### Transcriptional induction of salt stress-induced and MAPK-dependent marker genes is not affected by CPK3

**Figure 4:**
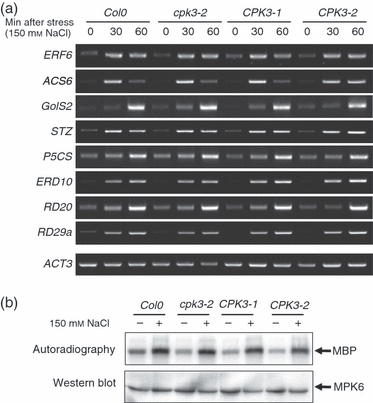
Crosstalk between MAPK and CPK3 kinase activities, and induction of marker genes, upon salt stress.(a) Salt-triggered induction of known salt stress-responsive marker genes was compared among the wild type (*Col-0*), *cpk3-2*, and the two *CPK3* overexpressor lines by RT-PCR, and compared with Actin (*ACT3*) as an internal control. Gene identifiers and sequences of the used primers are listed in Table S1. Fourteen-day-old seedlings were treated with 150 mm NaCl for the indicated time periods, as described in the Experimental procedures.(b) Salt-triggered activation of MPK6 in wild type (*Col-0*), *cpk3-2* knock-out, and two independent *CPK3* overexpressor lines towards myeline basic protein (MBP) as generic substrate. Kinase activities were measured in immunocomplex kinase assays upon salt treatment of 14-day-old seedlings for 15 min, as described in the Experimental procedures.

The observed nuclear localization of CPK3 suggests a role in the transcriptional regulation of the stress response. This is known for MAPK pathways, which predominantly target transcriptional responses in animals (Whitmarsh, 2007) and plants, considering the gene expression studies (Qiu *et al.*, 2008b) and identified MAPK targets involved in transcriptional regulation (Qiu *et al.*, 2008a). To look further downstream into this pathway, we analysed transcriptional regulation of salt stress-induced and MAPK-dependent marker genes in *cpk3* knock-out and overexpressor lines. A total of 22 target genes known to be regulated in the salt stress response, based on published microarray data (Kreps *et al.*, 2002; Seki *et al.*, 2002; Taji *et al.*, 2004), were studied by semi-quantitative RT-PCR before, and 30 and 60 min after, salt stress treatment in wild-type plants (Col-0), the *cpk3* mutant (*cpk3-2*), and the two *CPK3* overexpressor lines *CPK3-1 and CPK3-2*. Figure 4a shows the results for eight genes involved in ethylene signalling and biosynthesis (*ERF6* and *ACS6*), synthesis of the compatible solutes galactinol (*GolS2*) and proline (*P5CS*), transcriptional regulation (*STZ*), and general stress response (*ERD10*, *RD20* and *RD29a*). It turned out that all of the selected genes were normally induced in all lines. Furthermore, the complete set of genes that we analysed in these studies included the Na^+^/H^+^ antiporters *NHX1* and *SOS1*, the Na^+^-induced K^+^ channel *KC1*, several genes involved in trehalose synthesis (*TPS1*, *TPS11* and *T6PP*) and proline catabolism (*PDH*), the ABA- and salt stress-responsive protein phosphatases *AHG3* and *PP2C*, and the general stress-response factors *ERD15* and *RD29b*. But also those typical salt stress marker genes were normally induced.

It is known that the MAPKs MPK4 and MPK6 are involved in the salt stress response in Arabidopsis (Ichimura *et al.*, 2000; Teige *et al.*, 2004), and that those two MAPKs are regulated by the upstream MAPK kinase MKK2 (Teige *et al.*, 2004). Moreover, *mkk2* mutants show a phenotype similar to *cpk3* on salt media, and the MKK2 pathway regulates a set of 127 target genes, including typical salt stress markers (Teige *et al.*, 2004). Therefore, we also tested the activation of MPK4 and MPK6 in *cpk3* knock-out and *CPK3* overexpressor lines in response to salt stress. However, no difference in MAPK activation could be detected for both, MPK6 (Figure 4b) and MPK4 (Figure S6a) in immunocomplex kinase assays. *Vice versa*, CPK3 kinase activity was also not affected in *MKK2* knock-out or overexpressor lines (Figure S6b), arguing against a direct crosstalk of CPK3 and salt stress-induced MAPKs.

### *cpk3* plants exhibit differences in protein phosphorylation under basal conditions and in response to salt stress

**Figure 5:**
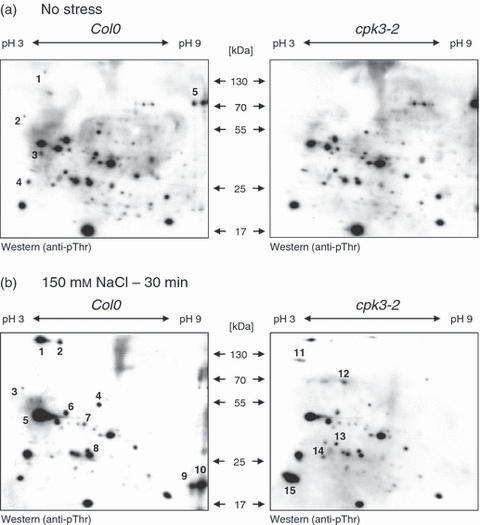
Comparison of threonine phosphorylation patterns in the roots of 6-week-old Col-0 and *cpk3-2*, hydroponically grown in half-strength Hoagland. (a) or half-strength Hoagland supplemented with 150 mm NaCl for 30 min before protein isolation (b). Spot patterns were obtained by western blot analysis with an anti *p*-Thr antibody. Numbers in the figures indicate spots showing different intensities between Col-0 and *cpk3-2* root material throughout three independent replications of the experiment.

The previous data, indicating that CPK3 activity has no influence on salt stress-dependent gene expression, gave rise to the question of how the salt-sensitive phenotype of the *cpk3* mutants could be explained. Therefore we searched for changes in protein phosphorylation patterns in wild-type and *cpk3* mutants in an unbiased proteomic approach. We analyzed phosphorylated threonine residues using a phosphoamino acid-specific antibody after 2D gel separation and western blotting of total proteins from salt-stressed and untreated plant extracts. Unfortunately, antibodies directed against phosphorylated serine residues proved not to be useful in these studies. Considering the important role of roots for salt stress adaptation, and the strong expression of CPK3 in this tissue (Figure 2a), we focused our studies on root cells isolated from a hydroponic culture of 6-week-old plants, and compared phosphorylation patterns of proteins in wild-type (Col-0) and *cpk3* knock-out plants (*cpk3-2*) before and 30 min after the application of salt stress. All experiments were carried out three times with identical results. More than 300 protein spots could be detected reproducibly after 2D gel separation of 100 μg of total protein by Coomassie staining, after blotting on polyvinylidene difluoride (PVDF) membranes. Some differences in threonine phosphorylation were already visible between the wild type and the *cpk3* mutant without stress conditions (Figure 5a). This result is consistent with the observed constitutive activity of CPK3 in roots and leaves, as shown in Figures 1b and S2. In the corresponding Coomassie stains, no significant differences in total protein spots could be detected (Figure S7), indicating that the observed differences are the result of post-translational modifications, and not to major differences in protein expression. The reproducibly observed changes are indicated by the numbers in Figure 5a,b. In summary, five differences (either new spots or strongly enhanced intensities) were detectable without stress between the wild type and the *cpk3* mutant (Figure 5a), and 15 differences appeared 30 min after the salt stress treatments (Figure 5b). In order to uncover CPK3 targets from this approach an identification of differentially phosphorylated proteins by mass spectrometry was performed. However, this approach was limited by two facts. First, only threonine phosphorylation could be detected, but serine phosphorylation is generally much more abundant. Second, the unambiguous identification of the protein spots corresponding to the signals from the western blot was extremely difficult in these complex samples. Therefore, we extended the proteomic approach towards the identification of potential CPK3 targets in microsomal fractions, considering the fact that CPK3 turned out to be membrane associated to a significant degree. Using radioactive [γ-^32^P]ATP and recombinantly expressed CPK3 for the Ca^2+^-dependent phosphorylation of microsomal fractions, we observed distinct CPK3-specific and Ca^2+^-dependent signals, without a significant background, in three independent experiments (Figure S8a); therefore, we proceeded to perform these experiments using radioactive and cold ATP in parallel, and to identify differentially phosphorylated spots after 2D gel separation by mass spectrometry (Figure S8b). From the 15 spots analysed we were able to unambiguously identify 78 proteins with very high confidence level (Table S2). In order to filter this data set further for potential CDPK targets, we analysed the proteins for a significant over-representation of known CDPK phosphorylation sites. We developed an algorithm that searches for the presence of five known CDPK consensus sites, and calculates their over-representation in comparison with the entire Arabidopsis proteome, also including a weighting reflecting the stringency of the defined consensus site. We termed the resulting figure a *P*-score, and the top 28 proteins with a *P-*score ≥ 4 were selected as potential CPK3 targets (Table 1). As expected, 20 of them are known membrane or membrane-associated proteins, and notably 13 of them are listed as phosphorylated proteins in the Phosphat database (http://phosphat.mpimp-golm.mpg.de/db.html).

**Table 1:** Potential CPK3 targets identified via 2D gel phosphoproteomics. Documented membrane association is indicated in bold. The *P*-score is described in the Experimental procedures

AGI	Annotation	*P*-score	References
AT2G30870	**ERD13, glutathione-*S*-transferase PHI 10**	32	Ryu *et al.* (2009), Carter *et al.* (2004) and Marmagne *et al.* (2007)
AT2G45820*	**Remorin, putative DNA-binding protein, salt-induced**	22	Benschop *et al.* (2007), Nelson *et al.* (2006) and Kreps *et al.* (2002)
AT1G04690	**KAB1, KV-BETA1 (potassium channel beta subunit)**	18	Maathuis (2006)
AT3G18240*	**Unknown protein, phosphorylated**	18	de la Fuente van Bentem *et al.* (2008)
AT1G53070*	**Legume lectin family protein, cell wall asscociated**	15	
AT1G09210*	**CRT2, Calreticulin 2; calcium ion binding**	13	Jiang *et al.* (2007)
AT1G56340*	**CRT1, Calreticulin 1; calcium ion binding**	12	Jiang *et al.* (2007)
AT5G38480*	**GRF3, 14-3-3 PSI, overexpression confers salt tolerance**	9	Marmagne *et al.* (2007) and Du *et al*. (2008)
AT3G02750*	**PP2C, protein phosphatase 2C family, group E**	9	Bassel *et al.* (2008)
AT5G19440	**CAD, Cinnamyl-alcohol dehydrogenase, putative**	9	Marmagne *et al.* (2007)
AT3G18820	**RAB71, (Arabidopsis RAB GTPase homolog G3F); GTP binding**	8	Marmagne *et al.* (2007)
AT2G02990	**RNS1, (Ribonuclease 1); cell wall/plasma membrane associated**	8	
AT1G02090*	CSN7, MAP kinase kinase; subunit of COP9 signalosome	7	
AT5G16050*	**GRF5, 14-3-3 UPSILON (general regularory factor 5)**	6	Marmagne *et al.* (2007)
AT2G43940	Thiol methyltransferase, putative, chloroplast envelope	6	
AT5G07340	**Calnexin, putative, vacuolar**	6	Schmidt *et al.* (2007)
AT1G28200	FIP1 (FH interacting protein 1), VirF-interacting protein FIP1	5	
AT1G78300	**GRF2, 14-3-3 OMEGA, (general regularory factor 2)**	5	Chang *et al.* (2009)
AT3G06300	**P4H-2, (prolyl-4-hydroxylase, isoform 2); endomembrane system**	5	
AT1G77120*	ADH1, (alcohol dehdrogenase 1); salt stress-responsive	5	de la Fuente van Bentem *et al.* (2008) and Jiang *et al.* (2007)
AT5G67500	**VDAC2 (voltage-dependent anion channel 2), seedling development**	5	Yan *et al.* (2009) and Lee *et al.* (2009)
AT3G01280	**VDAC1 (voltage-dependent anion channel 1)**	5	Lee *et al.* (2009)
AT1G74020	**SS2 (STRICTOSIDINE SYNTHASE 2); strictosidine synthase**	5	Marmagne *et al.* (2007)
AT4G17720*	RNA recognition motif (RRM)-containing protein, phosphorylated	5	de la Fuente van Bentem *et al.* (2006)
AT4G18800	**RABA1D (Arabidopsis RAB GTPase homolog A1D);**	5	Marmagne *et al.* (2004)
AT3G61990	*O*-methyltransferase family 3 protein, cytosolic	4	
AT1G22280*	PAPP2C, phytochrome-associated protein phosphatase 2C; group F	4	de la Fuente van Bentem *et al.* (2008) and Phee *et al.* (2008)
AT3G15260*	PP2C, protein phosphatase 2C family, group F	4	

* Phosphorylated peptides listed in the Phosphat 3.0 database (http://phosphat.mpimp-golm.mpg.de/db.html).

## Discussion

We have provided a detailed functional analysis of the calcium-dependent protein kinase CPK3, covering protein function and subcellular localization, as well as analysis of knock-out and overexpressor plants, and the identification of potential targets. We found that CPK3 kinase activity is triggered by salt and other stresses after transient overexpression in the protoplast system, but that the endogenous kinase appeared to be constitutively active in roots and leaves *in planta*. This apparent discrepancy might be caused simply by the strong difference in the expression level, which could affect low basal Ca^2+^ signals. Another explanation could be that large quantities of Ca^2+^ are released from the cell wall/apoplast during the preparation of the protein extracts from plant tissues, but not from protoplasts. Nevertheless, *cpk3* knock-out mutants displayed a salt-sensitive phenotype, whereas *CPK3* overexpression improved salt tolerance, and this effect correlated with *CPK3* expression levels, as revealed by RT-PCR. Only sparse functional and *in planta* data on this particular CDPK have been published so far. Dammann *et al.* (2003) showed a cytosolic/nuclear localization of CPK3-GFP fusion proteins in roots, and Mori *et al.* (2006) showed that *cpk3 cpk6* double knock-out plants displayed altered responses of vacuolar potassium channels in leaf guard cells in response to ABA, but that neither a direct phosphorylation of the channel itself nor a phenotype of the knock-out plants in response to stress was exhibited. The phenotype we observed for *cpk3* mutants resembled that of MAPK mutants under similar stress conditions (Teige *et al.*, 2004; Qiu *et al.*, 2008b), which prompted us to extend the functional analysis of CPK3 in the salt stress response of Arabidopsis towards an analysis of the potential crosstalk between CPK3- and MAPK-dependent signalling, focussing on MPK4 and MPK6, which are the major players in the MAPK-mediated salt stress response.

Crosstalk between Ca^2+^ and MAPK signalling is well known for animal cells, were Ca^2+^ signals and calmodulines (CaMs) regulate the Ras/Raf/ERK-MAPK pathway (Agell *et al.*, 2002; Rozengurt, 2007), but this general question has almost not been addressed in plants. Ludwig *et al.* (2005) reported ethylene-mediated crosstalk between CDPK and MAPK signalling, demonstrating that elevated CDPK activities compromised stress-induced MAPK activities by the overexpression of a truncated, and thereby deregulated, tobacco CDPK. This inhibition required ethylene synthesis and perception. In contrast, very recently Boudsocq *et al.* (2010) reported that CDPKs and MAPK cascades act differentially in regulatory programmes, in response to microbe-associated molecular patterns. In line with this latter report, our analysis provides no evidence for the interference of CPK3 kinase activity with MPK4 and MPK6 activities in response to salt stress, notably in both directions. The induction of salt stress-induced and MAPK-dependent marker genes (Teige *et al.*, 2004; Qiu *et al.*, 2008b), performed in a *cpk3* knock-out and two independent *CPK3* overexpressor lines, also indicated that MAPK target genes and salt-triggered transcriptional induction of response genes are not affected by CPK3. From these data we propose that CPK3 and MAPKs act independently, and in parallel, in the salt stress response. This is not necessarily valid for other stress responses, i.e. for pathogen stress, as Boudsocq *et al.* (2010) showed that transiently expressed and deregulated CPK3 is able to induce the flagellin-dependent *NHL10* promoter in mesophyll protoplasts. Also in animal cells, quite different forms of crosstalk between Ca^2+^ and MAPK signalling have been published (Agell *et al.*, 2002; Rozengurt, 2007).

The lack of transcriptional response of known salt stress-regulated marker genes in *CPK3* knock-out or overexpressor lines in response to salt stress raised the question of how the salt-sensitive phenotype could be explained. In yeast, at least two signalling pathways are involved in the regulation of ion homeostasis and osmotic adjustment. The Ca^2+^-dependent phosphatase calcineurin regulates the expression of ion transporters like *ENA1*, the major Na^+^ efflux pump in the plasma membrane, and the MAPK Hog1 is required for transcriptional adaptation (Hohmann, 2002; Matsumoto *et al.*, 2002). Hog1 also regulates the activities of the Nha1 Na^+^/H^+^ antiporter and the Tok1 potassium channel by phosphorylation (Proft and Struhl, 2004). This dual role of Hog1 in yeast osmostress adaptation appears to have split in the salt stress response in plants. Here, the MAPK pathway seems to be mainly responsible for the transcriptional induction of genes required for long-term adaptation, whereas CPK3 seems to regulate membrane-associated target proteins by phosphorylation in the immediate response.

So far, only a few proteomic studies of the plant salt stress response have been performed, and they have focused on the long-term changes in protein levels. Salt stress first causes a transient suppression of *de novo* protein synthesis in yeast and plant cells (Teige *et al.*, 2001; Ndimba *et al.*, 2005), and visible changes in total protein patterns can only be observed after several hours (Ndimba *et al.*, 2005; Jiang *et al.*, 2007). It is clear that an additional, immediate mechanism of adaptation is required to enable plant survival in an acute stress situation. Only one study has addressed the rapid changes in protein phosphorylation in response to salt stress in plants so far, and has reported the multiple phosphorylation of plasma membrane aquaporins (Prak *et al.*, 2008), which were also identified in membrane fractions after 3 days of salt stress (Hsu *et al.*, 2009).

Our proteomic search for potential CPK3 targets in microsomal fractions revealed no overlap with those two latter studies, but identified a number of proteins with known regulatory functions in signalling and stress response, including salt stress. The identification of three different 14-3-3 proteins, three PP2C-type protein phosphatases, two RAB GTPases, one protein kinase and several ion channels highlights the important function of membrane-associated signalling events, and is in line with recent proteomic studies of plant plasma membranes. The concept that specific lipid micro-environments – known as lipid rafts – cause a spatial organization of protein complexes with important functions in signalling was developed for animal cells years ago (Simons and Ikonen, 1997; Simons and Toomre, 2000), but only recently has evidence for this phenomenon been obtained for plant cells (Morel *et al.*, 2006; Marmagne *et al.*, 2007). Notably, a number of different CDPKs have been identified in proteomic studies of detergent-resistant plasma membranes from tobacco (Morel *et al.*, 2006), and CPK9, CPK32 and CPK3 have been detected at plasma membranes in Arabidopsis (Nelson *et al.*, 2006). CPK7, CPK10 and the DNA-binding remorin protein (At2g45820), which we identified with a high score as a potential CPK3 target in our analysis, were also found as differentially phosphorylated proteins in early elicitor signalling (Benschop *et al.*, 2007). Moreover, this particular remorin is transcriptionally induced in response to salt stress (Kreps *et al.*, 2002). Co-localization of the protein kinase and its targets would obviously favour fast and efficient signal transduction, particularly if the activating signal is extremely transient and locally restricted, as is the case for Ca^2+^ signals (Bootman *et al.*, 2001). Accordingly KAB1, a potassium channel subunit, and the two voltage-dependent anion channels (VDACs), would present bona fide CPK3 targets. KAB1 responds to cyclic guanyl monophosphate (cGMP) signals, which are known to occur in response to salt or osmotic stress in plants (Maathuis, 2006). The vacuolar two-pore K^+^ channel TPK1 was reported to be phosphorylated at a 14-3-3 binding site (Latz *et al.*, 2007), and this site proved recently to be phosphorylated by different CDPKs, including CPK3 (Latz *et al.* submitted). The early dehydration-induced gene *ERD13*, a glutathione-*S*-transferase, has been localized to the vacuole (Carter *et al.*, 2004) and plasma membrane (Marmagne *et al.*, 2007), and Ryu *et al.* (2009) showed that its overexpression conferred salt tolerance, and that its downregulation caused an increased salt sensitivity in Arabidopsis. In a functional screen for genes conferring salt tolerance to Arabidopsis, Du *et al.* (2008) isolated the 14-3-3 protein GRF3, which also localizes to the plasma membrane (Marmagne *et al.*, 2007). 14-3-3 proteins do not only bind to phosphorylated proteins, notably at sites that are phosphorylated by CDPKs (Ishida *et al.*, 2008), and also to CDPKs (Camoni *et al.*, 1998), they are also known to be phosphorylated themselves (Aitken, 2002). A recent proteomic profiling of tandem affinity purified 14-3-3 protein complexes using GRF2 as bait revealed many channels involved in ion transport and hormone signalling, including the brassinolide receptors BRI1 and BAK1 (Chang *et al.*, 2009). Considering that ERD13 has been isolated as an interacting partner of BAK1 in a yeast two-hybrid screen by Ryu *et al.* (2009), a new picture emerges where CPK3 could be a regulator of the interactions taking place in membrane-associated protein complexes.

The identification of three PP2C-type protein phosphatases, including one with predicted *N*-myristoylation and membrane localization (At3g02759), gives the first hints about negative regulators of CPK3. The importance of negative regulation is well known from ABA signalling, and recently a pathway where three PP2C phosphatases regulate the protein kinase SnRK2.6/OST1 has been described by Fujii *et al.* (2009). The observation that the aforementioned PP2C has also been identified in a screen for germination-specific transcripts (Bassel *et al.*, 2008) adds further evidence to the functional context of CPK3 and the salt-sensitive germination phenotype.

CPK3 has been localised to the cytoplasm and the nucleus previously by Dammann *et al.* (2003), but a partial membrane association of CPK3 is also visible in their work. In line with these results, some cytoplasmic targets of CPK3 also appear in our proteomics analysis, and others have been reported recently by Rietz *et al.* (2010). We found a substantial level of CPK3 associated with different cellular membranes, depending on its *N*-myristoylation. We could clearly observe co-localization of CPK3-YFP with a vacuolar and a plasma membrane marker in infiltrated leaves, and a biochemical fractionation of endogenous CPK3 by two-phase partitioning confirmed its localization at the plasma membrane. Notably, this partitioning does not distinguish between remaining plasma membranes and other membranes in the lower phase, thus leaving the evidence for vacuolar localization of CPK3 based on the co-localization of the YFP fusion proteins. A similar complex localization pattern as found for CPK3 has been described for CPK32, which harbours *N*-myristoylation and palmitoylation sites (Choi *et al.*, 2005). Notably CPK3 can only be *N*-myristoylated, but not palmitoylated, because of the lack of cysteine residues in its very N terminus. This might explain that CPK3 is not exclusively restricted to one particular membrane, as is the case for many of the *N*-myristoylated and palmitoylated CDPKs. CPK32 phosphorylates the bZIP transcription factor ABF4, a regulator of ABA-responsive gene expression, and thereby affects abiotic stress tolerance, including salt stress. ABF4 was also found to be phosphorylated by CPK4 and CPK11, two CDPKs that lack N-terminal acylation motifs, resulting in cytoplasmic and nuclear localization (Dammann *et al.*, 2003), with similar effects on abiotic stress tolerance and germination (Zhu *et al.*, 2007). A nuclear/cytoplasmic localization has also been reported for several plant MAPKs, including Arabidopsis MPK4 and MPK6 (Schweighofer *et al.*, 2007). The activation of gene expression through MAPK cascades involves dynamic changes of their subcellular localization, also reflecting the localization of their potential targets (Lee *et al.*, 2004). In this context, the observed *N*-myristoylation-dependent membrane localization of CPK3 would provide a molecular basis for the different tasks of CDPK and MAPK pathways in a plant’s salt stress response.

## Experimental procedures

### Plant cultivation

*Arabidopsis thaliana* (ecotype Columbia, Col-0) seeds were surface sterilized using the vapour-phase method (Clough and Bent, 1998). Sterile cultivation of was performed on half-strength MS agar plates, supplemented with 50 μg ml^−1^ kanamycin when required. Plants were grown under 16 h of light with 120 μmol m^−2^ s^−1^ light intensity at 25°C. Seeds were stratified by incubation in the dark at 4°C for 2 days prior to placing them in the light. Germination assays were performed on agar plates containing quarter-strength Hoagland medium supplemented with 150 mm NaCl. Germination rates (per seeds) were scored after 6 days. The hydroponic cultivation of Arabidopsis plants was performed in half-strength Hoagland medium according to the method described by Tocquin *et al.* (2003).

### Arabidopsis suspension culture and protoplast transformation

Arabidopsis protoplasts were prepared from a root suspension culture (Mathur and Koncz, 1998), and transient expression assays were performed as described by Cardinale *et al.* (2002). The open reading frame of CPK3 was cloned into the plant expression vector pRT100 (Topfer *et al.*, 1987) and fused to a triple HA epitope at the C-terminal end.

### Analysis of cpk3 T-DNA insertion lines

*CPK3* T-DNA insertion lines (Figure S1) were obtained from the Salk Arabidopsis Insertion Library (http://signal.salk.edu) (Alonso *et al.*, 2003). Kanamycin-resistant plants were propagated as individual lines and analysed by PCR, using gene- and T-DNA-specific primers to identify the positions of the T-DNA insertion. Further analysis of *CPK3* expression was performed by semiquantitative RT-PCR analysis and western blotting.

### Generation of CPK3 overexpressing lines

The *CPK3* coding region was cloned into the binary plant expression vector pBIN19 (Datla *et al.*, 1992) under the control of the cauliflower mosaic virus (CaMV) *35S* promoter and transformed as YFP-epitope-tagged versions into Col-0 wild-type plants using the floral-dip method (Clough and Bent, 1998). Transformed plants were selected on kanamycin-containing media, and *CPK3* expression was analysed by RT-PCR.

### Molecular cloning and construction of expression vectors

Open reading frames of studied MAPKs, CDPKs and TPK1 were amplified either from RAFL full-length cDNA clones (http://www.brc.riken.jp/lab/epd/Eng/species/arabidopsis.shtml) or from a cDNA library (Minet *et al.*, 1992), introducing an *Nco*I or *Apa*I restriction site at the 5′ end and a *Not*I restriction site in front of the stop codon. The *Not*I restriction site at the 3′ end was used to fuse a triple HA epitope, YFP or mCherry tag.

### Expression and purification of GST-fusion proteins

*Escherichia coli* strain BL-21 codon plus (Stratagene, http://www.stratagene.com) was transformed with expression constructs, cloned into the pGEX4-T1 vector (Amersham, http://www.gehealthcare.com). The growth of bacteria and isolation of recombinant GST fusion proteins were performed according to the method described by Matsuoka *et al.* (2002) using glutathione sepharose TM 4B (Amersham), following the manufacturer’s instructions. Proteins were eluted with 33 mm reduced glutathione, 250 mm NaCl, 0.5% Triton X-100 in Tris-buffered saline (TBS) Tween, which was changed to kinase buffer (20 mm HEPES, pH 7.5, 15 mm MgCl_2_, 8 mm EDTA, 1 mm DTT) by using PD10 columns (Amersham).

### Protein extracts from plant material

Proteins from all plant materials were extracted in protein extraction buffer (Bogre *et al.*, 1999), containing 25 mm Tris, pH 7.8, 75 mm NaCl, 10 mm MgCl_2_, 15 mm EGTA, 1 mm DTT, 1 mm NaF, 0.5 mm NaVO_3_, 15 mmβ-glycero-phosphate, 15 mm*p*-nitrophenyl-phosphate, 0.1% Tween 20, 0.5 mm phenylmethylsulfonyl fluoride, 5 μg ml^−1^ leupeptin, and 5 μg ml^−1^ aprotinin. Usually 200 mg of leaf material were ground in a 1.5-ml reaction tube in 200 μl of protein extraction buffer together with sea sand. Extracts were further clarified by centrifugation at 16 000 ***g*** for 10 min at 4°C.

### *In vitro* testing for *N*-myristoylation

The coding regions of *CPK2* and *CPK3* were cloned into pBAT (Annweiler *et al.*, 1991) after the introduction of an *Apa*I restriction site at the 5′ end, and a *Not*I site at the 3′ end. *N*-myristoylation of proteins was tested as described previously (Lu and Hrabak, 2002; Benetka *et al.*, 2008) after coupled *in vitro* transcription/translation in a cell-free system (TNT Coupled Wheat Germ Extract System; Promega, http://www.promega.com). A 2-μg portion of plasmid template was linearized (*Not*I) and *in vitro* translated either in the presence of 10 μCi of l-[^35^S]methionine (1175 Ci mmol^−1^, for total protein labelling; Perkin Elmer, http://www.perkinelmer.com), or 50 μCi of [9,10-^3^H]-labelled myristic acid (60 Ci mmol^−1^; American Radiolabeled Chemicals, http://www.arc-inc.com). Reaction products were separated on 10% (w/v) SDS-polyacrylamide gels, and incubated with autoradiography intensifier prior to detection on X-ray film.

### Immunocomplex kinase assays

Immunocomplex kinase assays were performed according to Teige *et al.* (2004) using peptide-specific antibodies against CPK3 and the Arabidopsis MAPKs MPK4 and MPK6, as described by Teige *et al.* (2004). The procedure and the assay conditions are described in detail in Appendix S1.

### Infiltration of tobacco leaves

*Nicotiana tabaccum* leaves were used for infiltration, as described in Bucher *et al.* (2003). YFP fusions of the coding regions of wild-type CPK3 and the corresponding G_2_A mutant, YFP alone, TPK1 and CPK9 were cloned as *Kpn*I–*Sac*I fragments in the binary plant expression vector pBIN19. Proteins were transiently expressed under the control of the CaMV *35S* promoter after infiltration of tobacco leaves with *Agrobacteria*. The pictures were taken 2 days after infiltration of developing young leaves using a Zeiss Axioplan laser scanning confocal microscope.

### Two-phase separation for plasma membrane isolation

Plasma membranes were isolated in an aqueous two-phase system according to Santoni (2007), with minor modifications. A detailed description of the procedure and the composition of the buffers can be found in Appendix S1.

### RNA isolation from Arabidopsis leaves and reverse-transcription PCR

Leaves from 2-week-old Arabidopsis plants were frozen in liquid nitrogen and 100 mg of leaf material was processed per sample. RT-PCR was carried out as described in detail by Doczi *et al.* (2007). Briefly, plant material was mixed with 130 μl RNA extraction buffer (1% SDS, 10 mm EDTA, 200 mm sodium acetate, pH 5.2), 130 μl phenol (pH 4.0) and sea sand, and then ground. RNA was subsequently extracted with phenol/chloroform/iso-amylalcohol and digested with RNAse-free DNAse (RQ1 DNAse; Promega). The concentration and purity of RNA was determined spectrophotometrically at 260 and 280 nm. A 2-μg portion of RNA was used for reverse transcription with M-MLV reverse transcriptase (Promega). PCR amplification of target genes was performed with GoTaq^©^ DNA polymerase (Promega), and products were separated on 1.5% agarose gels. A complete list of all primers and gene identifiers for the target genes is shown in Table S1.

### 2D gel electrophoresis and western blotting of Arabidopsis root extracts

Total root protein extracts were prepared using the phenol protein extraction method, according to Isaacson *et al.* (2006). A 100-μg portion of protein was separated by 2D gel electrophoresis as described in detail in Appendix S1, and then transferred to PVDF membranes. Detection of protein phosphorylation on threonine residues was performed using an anti-phosphothreonine antibody (Cell Signalling Technology, http://www.cellsignal.com).

### Enzymatic digest, LC-MS/MS analysis and data analysis

Coomassie-stained gel spots were excised from the 2D gel and used for nano-electrospray LC-MS/MS investigations after a destaining tryptic digest. After separation on a C18 reverse phase column, mass spectra were obtained in an LTQ (Thermo, http://www.thermofisher.com) linear ion trap mass spectrometer, and MS/MS spectra were interpreted in Mascot 2.2 (Matrix Science, http://www.matrixscience.com) and Bioworks 3.3 (Thermo). The database used for the search was the TAIR9 protein database (ftp://ftp.arabidopsis.org/home/tair/Sequences/blast_datasets/TAIR9_blastsets/TAIR9). Further details on protein identification can be found in Appendix S1. To quantify the over-representation of CDPK consensus phosphorylation sites, we analysed the identified proteins for the presence of the following five overlapping CDPK phosphorylation motifs: [S]-X-[KRP], [RK]-X-X-[ST]-X-[KRP], [KR]-X-X-[ST], ϕ-X-[KR]-X-X-S-X-[KRP], or [RKHYCDE]-X-X-[KR]-X-X-S-X-X-[KR], see Huang *et al.* (2001), Cheng *et al.* (2002) and Hernandez Sebastia *et al.* (2004). Their over-representation was compared with the entire Arabidopsis proteome by summing up the probability for each motif to appear, as compared with the entire proteome, and normalising to the protein length. We termed the *P*-score of a protein X of length *l* as:
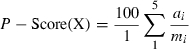


Where the motif *i* occurs *a*_*i*_ times in a given protein X, with the probability *m*_*i*_ to occur by chance in the total Arabidopsis proteome.

## SUPPORTING INFORMATION

**Figure S1.** Characterization of three independent T-DNA insertion lines obtained from the Salk Arabidopsis Insertion Library (http://signal.salk.edu).

**Figure S2.** Kinetics of CPK3 kinase activity in leaves and seedlings in response to salt stress.

**Figure S3.** Germination rates of *CPK3* overexpressor lines under salt stress conditions.

**Figure S4.** Localisation of wild-type CPK3 and CPK3 G_2_A mutants in leaves and chloroplast import.

**Figure S5.** Supporting material for Figure 3 (localization of wild-type CPK3 and CPK3 G_2_A mutants, including bright-field images).

**Figure S6.** Crosstalk between MAPK and CPK3 kinase activities, and salt-induced induction of marker genes.

**Figure S7.** Loading control for comparison of threonine phosphorylation patterns of the Col-0 and *cpk3-2* plants shown in Figure 4.

**Figure S8.** Phosphorylation of microsomal membranes by recombinant CPK3 and 2D gel separation for MS analysis.

**Table S1.** List of used oligonucleotides for cloning and PCR analysis.

**Table S2.** Identified proteins containing peptide sequences and scores.

**Appendix S1.** Detailed description of experimental procedures and materials.

Please note: As a service to our authors and readers, this journal provides supporting information supplied by the authors. Such materials are peer-reviewed and may be re-organized for online delivery, but are not copy-edited or typeset. Technical support issues arising from supporting information (other than missing files) should be addressed to the authors.
