# Supplementary figures and images for: The Ca2+-dependent protein kinase CPK3 is required for MAPK-independent salt-stress acclimation in Arabidopsis
Source: Plant J. 2010 Jun 15;63(3):484–98. doi: 10.1111/j.1365-313X.2010.04257.x (PMC2988408; doi:10.1111/j.1365-313X.2010.04257.x)

(a)

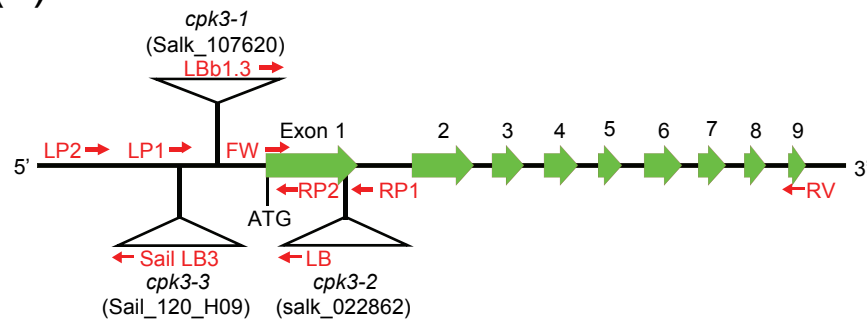

(b)

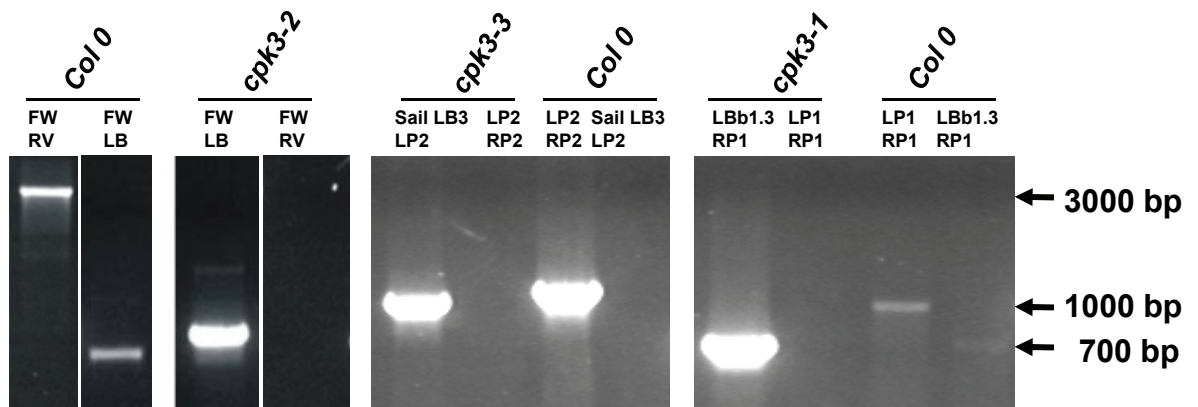

(c)

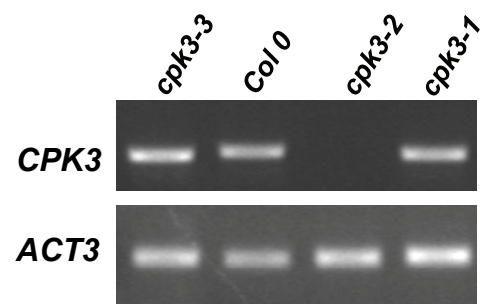

Supplement: Supplementary file 1 [file tpj0063-0484-SD1.pdf]

(a)

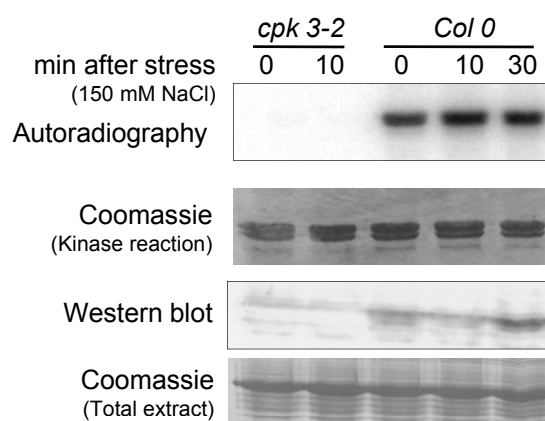

(b)

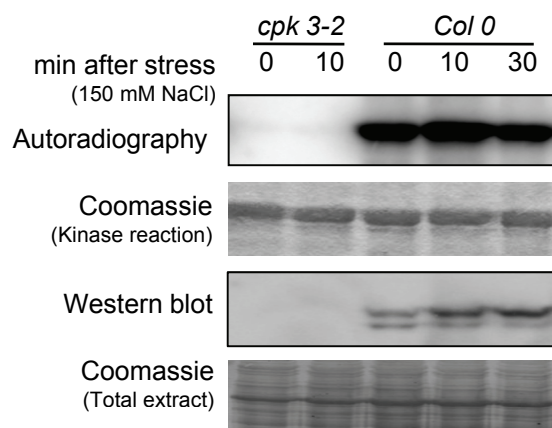

Supplement: Supplementary file 2 [file tpj0063-0484-SD2.pdf]

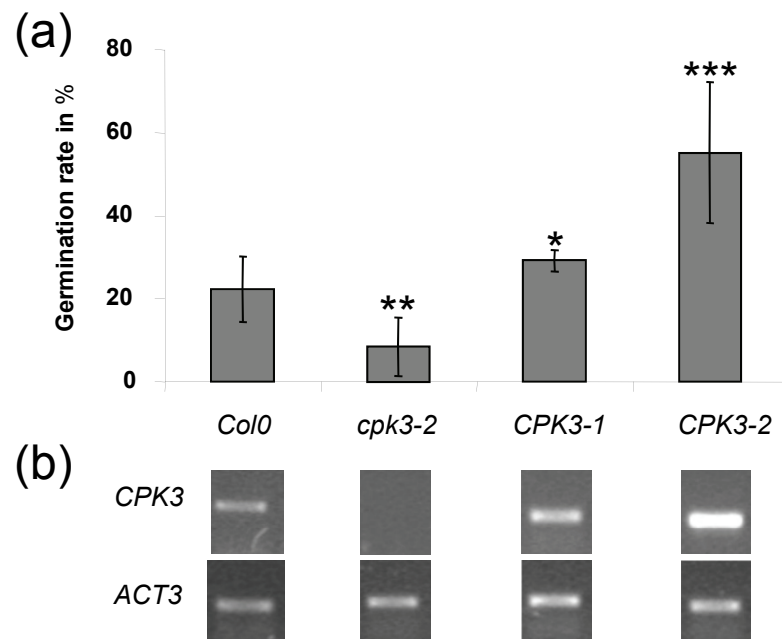

Supplement: Supplementary file 3 [file tpj0063-0484-SD3.pdf]

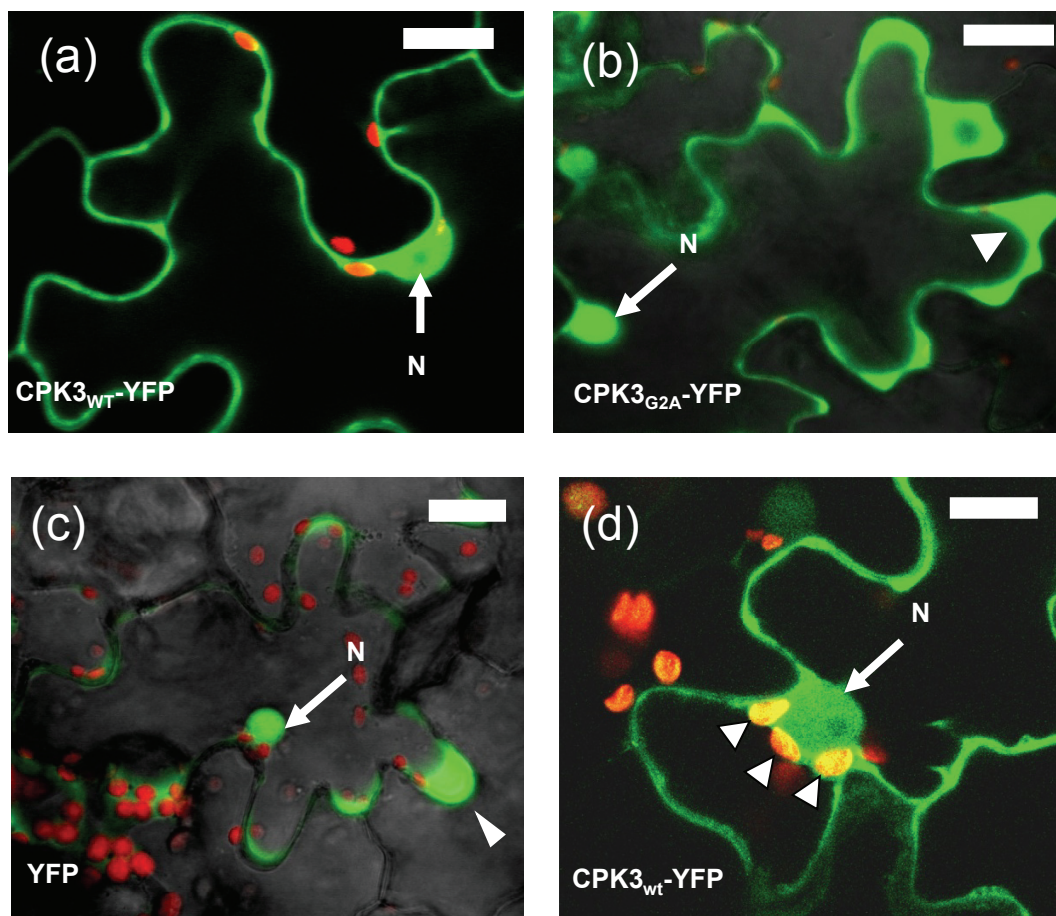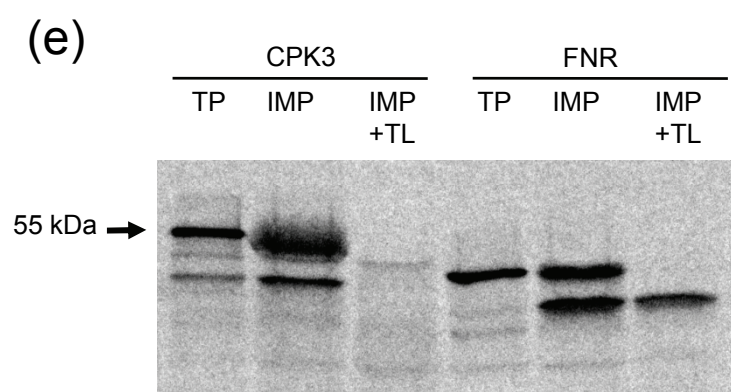

TP – translation product  
 IMP – import  
 TL – thermolysin digest

Supplement: Supplementary file 4 [file tpj0063-0484-SD4.pdf]

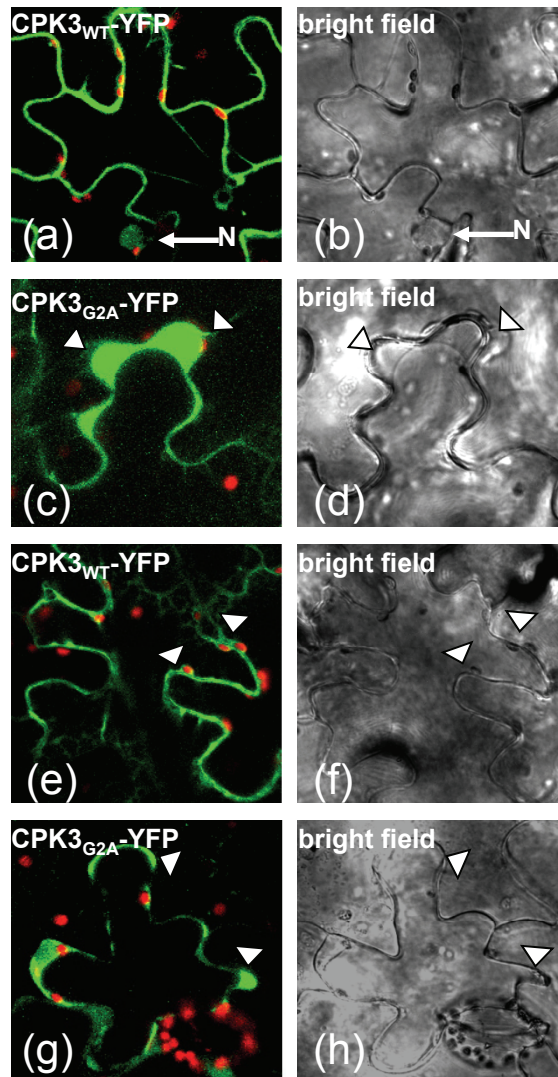

Supplement: Supplementary file 5 [file tpj0063-0484-SD5.pdf]

(a)

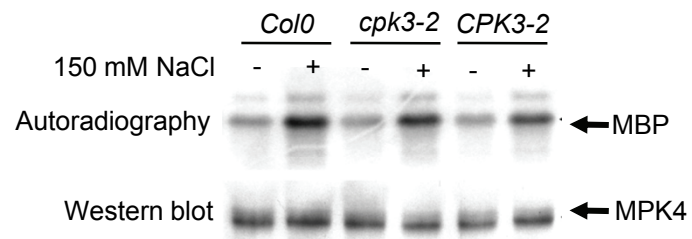

(b)

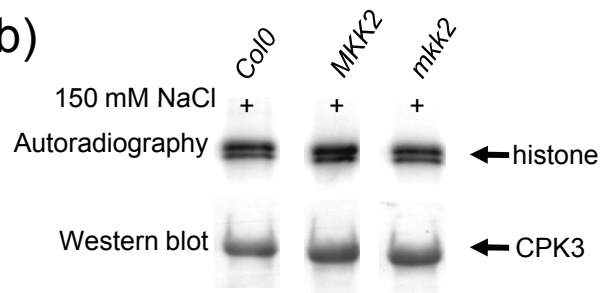

Supplement: Supplementary file 6 [file tpj0063-0484-SD6.pdf]

(a)

no stress

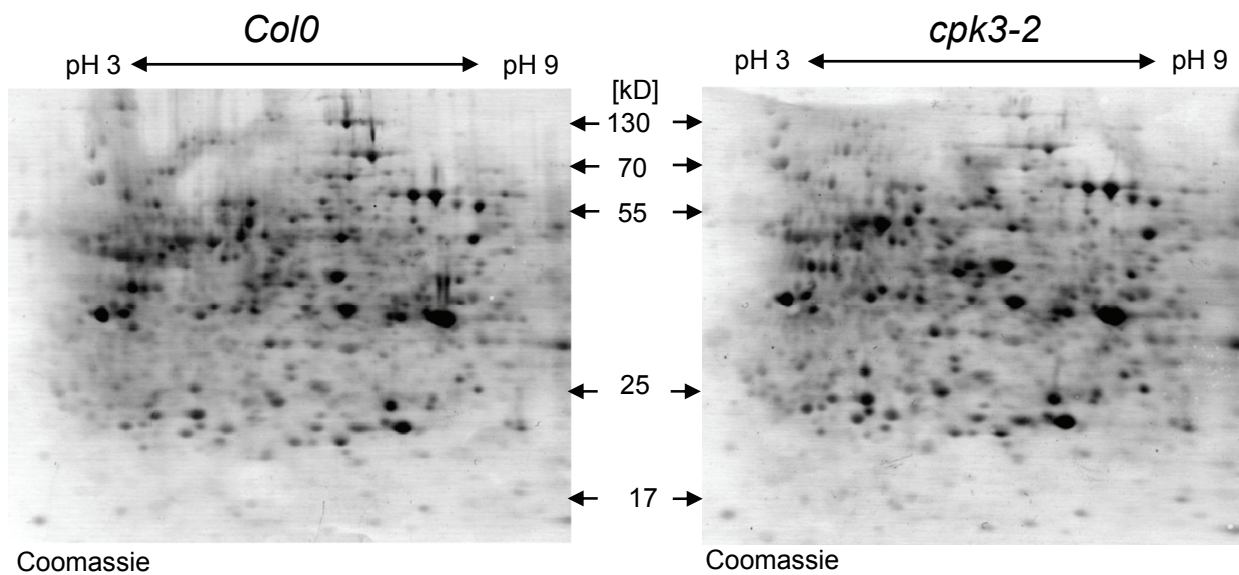

(b)

150 mM NaCl – 30 min

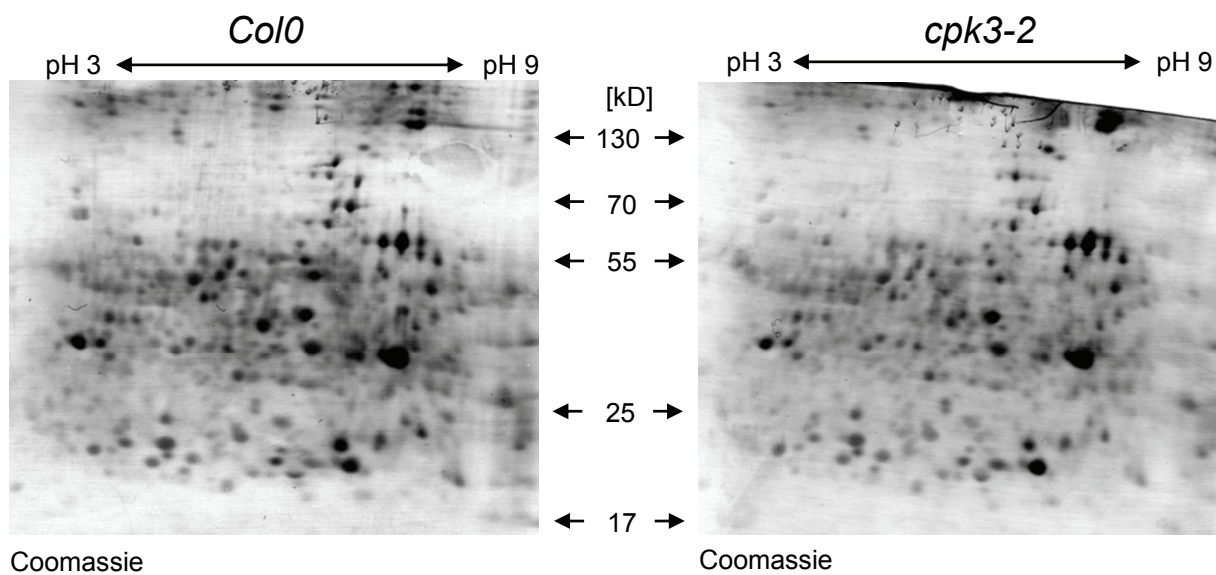

Supplement: Supplementary file 7 [file tpj0063-0484-SD7.pdf]

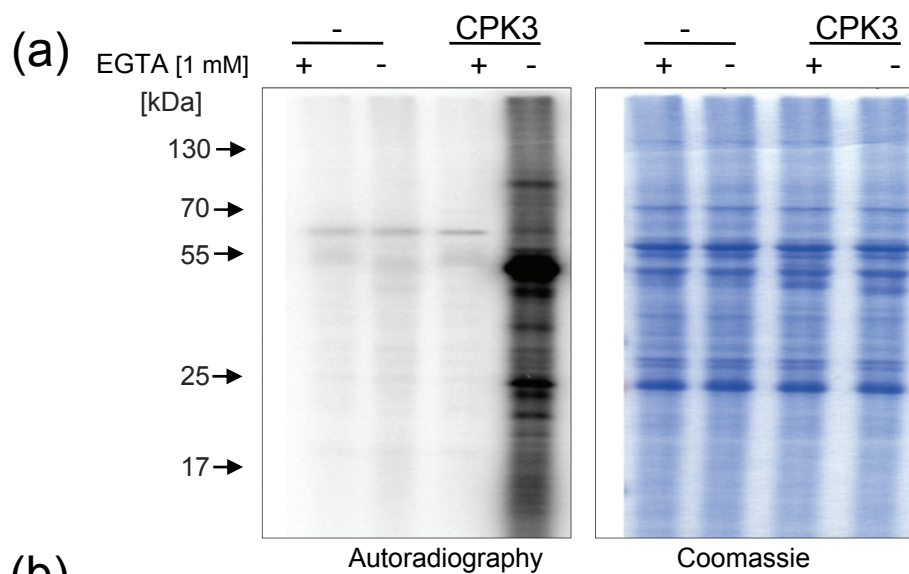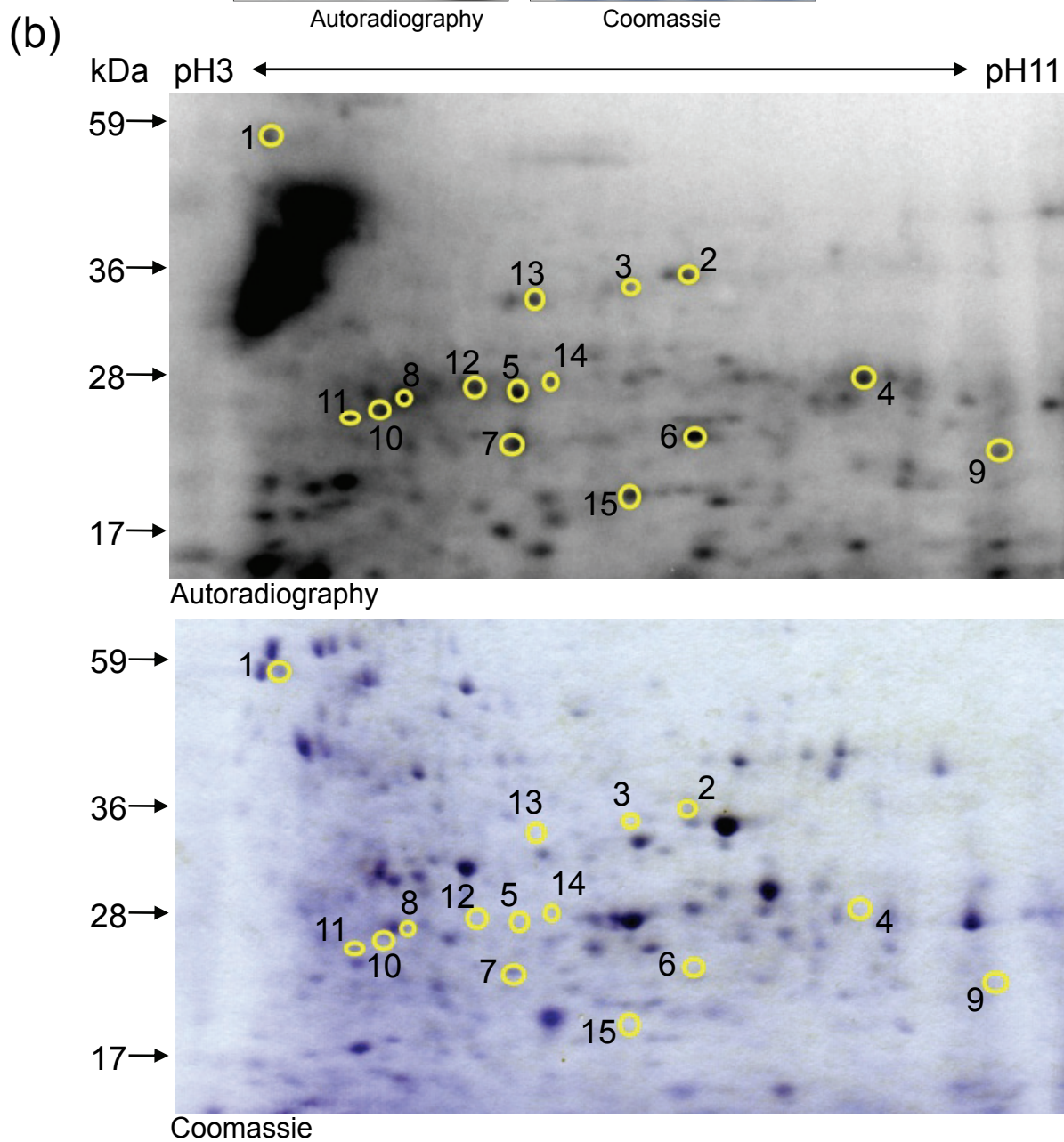

Supplement: Supplementary file 8 [file tpj0063-0484-SD8.pdf]
