# Supplementary material for: The Ca2+-dependent protein kinase CPK3 is required for MAPK-independent salt-stress acclimation in Arabidopsis
Source: Plant J. 2010 Jun 15;63(3):484–98. doi: 10.1111/j.1365-313X.2010.04257.x (PMC2988408; doi:10.1111/j.1365-313X.2010.04257.x)
Supplement: Supplementary file 9 [file tpj0063-0484-SD9.doc]

**Supplemental TABLE 1** Primers for mapping the T-DNA insertions and used for RT-PCR.

|  | **Primers for insertion mapping** |
| --- | --- |
| *Salk_LB* | 5'-CGC TGG ACC GCT TGC TGC AAC T-3' |
| *Salk_LBb1.3* | 5'-ATT TTG CCG ATT TCG GAA C-3' |
| *Sail-LB3* | 5'-TAG CAT CTG AAT TTC ATA ACC AAT CTC GAT ACA C-3' |
| *CPK 3-2 FW* | 5'-AAA AGG ATC CGG GCC CAT GGG CCA CAG ACA CAG CAA GTC CAA ATC CTC CG-3' |
| *CPK 3-2 RV* | 5'-TTT TGT CGA CCT AGC GGC CGC ACA TTC TGC GTC GGT TTG GCA CCA ATT CTG GAT TTC CC-3' |
| *CPK 3-1 RP1* | 5'-ACG AGG TAC GTG ACA CCA AAC-3' |
| *CPK 3-1 LP1* | 5'-TTG TGT CGA ACA AGT GGT TTG-3' |
| *Sail 120 H09 RP2* | 5'-GTA GGC TCC CTT CAA GTC CAC-3' |
| *Sail 120 H09 LP2* | 5'-CAT TGC CAG AAA AGC TGA AAC-3' |
|  |  |
|  | **RT-PCR oligos** |
| *ERF6-1* | 5'-CCG TTG CCT ACT ACT GCC ACC-3' |
| *ERF6-2* | 5'-GCA CTT TCT CAA CC ACC GTC-3' |
| *ACS6-1* | 5'-GAG CGG CGG CGC AAC CGG AG-3' |
| *ACS6-2* | 5'-CCA CCC TGT CAT TGT AAG AG-3' |
| *GolS2-1* | 5'-AAG GCT GTG TCG TGC GTG AG-3' |
| *GolS2-2* | 5'-GGC TTG GAT CCA GCT GCA CAG-3' |
| *STZ1-1* | 5'-ATG GCG CTC GAG GCT CTT AC-3' |
| *STZ1-2* | 5'-TCC TTC GTA GTG GCA CCG C-3' |
| *P5CS2-1* | 5'-CGT CGT CAA GGT TGG GAC TGC-3' |
| *P5CS2-2* | 5'-TCT AGC GAC AGA AGA GCG GC-3' |
| *ERD10-1* | 5'-TCT TCC TCT TCG AGT GAT GAA G-3' |
| *ERD10-2* | 5'-TCT CTT CCT CTC CAG TGG-3' |
| *RD20-1* | 5'-CCA AAA CCA TAC ATG GCA AGA GC-3' |
| *RD20-2* | 5'-TGA AAG CCA TCC AAA AGG ATC G-3' |
| *RD29a-1* | 5'-AGC ACC CAG AAG AAG TTG AAC ATC-3' |
| *RD29a-2* | 5'-CGT TAC ATC CTC TGT TCC AG-3' |
| *ACT3-1* | 5'-ATG GTT AAG GCT GGT TTT GC-3' |
| *ACT3-2* | 5'-AGC ACA ATA CCG GTA GTA CG-3' |
| *CPK3i-1* | 5'-AGA TGT TCG CCG TGA AGT CC-3' |
| *CPK3i-2* | 5'-ACG GAT GAT TTA GCA CTT CCG-3' |
