# Supplementary material for: The Ca2+-dependent protein kinase CPK3 is required for MAPK-independent salt-stress acclimation in Arabidopsis
Source: Plant J. 2010 Jun 15;63(3):484–98. doi: 10.1111/j.1365-313X.2010.04257.x (PMC2988408; doi:10.1111/j.1365-313X.2010.04257.x)
Supplement: Supplementary file 11 [file tpj0063-0484-SD11.doc]

# EXPERIMENTAL PROCEDURES

## *Immunocomplex Kinase Assays*

150-250 mg plant material (7 day-old seedlings or leaves/roots from hydroponic culture) were grinded in protein extraction (25mM TrisCl pH 7.5; 15mM MgCl_2_; 15mM EGTA; 75mM NaCl; 1mM DTT; 0.1% Nonidet P-40; 15mM β-glycerophosphate; 0.5mM Na_3_VO_3_; 1mM NaF; supplemented with Complete-EDTA free protease inhibitors from Roche, and clarified by centrifugation at 16,000g for 10min at 4°C. For one immunopreciptitation (IP) the total protein content was adjusted to 100µg and supplemented with 20µl protein A-Sepharose. The IP was performed for 1h with 3µl antibody at 4°C. Protein A-beads were washed three times with extraction buffer, once with wash buffer (50mM TrisCl pH 7.5; 5mM EGTA; 5mM EDTA; 0.1% Tween 20; 0.1% Nonidet P-40; 250mM NaCl; 5mM NaF; and finally once with kinase buffer (20mM HEPES pH 7.5; 15mM MgCl_2_; 1mM DTT; 1mM EGTA or 100µM CaCl_2_). CDPK kinase assays were carried out in the presence of 100µM CaCl_2_ using Histone IIIS (1µg Sigma Aldrich) as generic substrate. MAPK kinase assays were performed in the presence of 1mM EGTA using myelin basic protein (MBP, 1µg) as generic substrate. Reactions were stopped after 20min by addition of SDS-loading buffer and heating at 95°C. The reaction products were analyzed by SDS-PAGE, autoradiography and Coomassie Brilliant Blue R250 staining.

### Two-Phase Separation for plasma membrane isolation

Plasma membranes were isolated in an aqueous two-phase system according to Santoni (2007) with minor modifications. 10-15 g leaves were homogenized in a Waring blender with 100ml homogenization buffer (50mM Tris pH 8.0 adjusted with MES, 400mM sorbitol, 20mM EDTA, 20mM EGTA, 50mM NaF, 15mM β-glycerophosphate, 1mM Na-orthovanadate, 10mM ascorbic acid, 5mM DTT, supplemented with EDTA-free Complete protease inhibitor (ROCHE; www.roche.com). The homogenate was filtered through two layers of miracloth (CALBIOCHEM; www.merck-chemicals.de/life-science-research) and centrifuged for 20min at 10,000g. The supernatant was centrifuged at 100,000g for 1h and the obtained microsomal pellet was resuspended in 4.5ml of microsome resuspension buffer and added to an 18-g phase system. After thorough mixing and centrifugation, primary upper and lower phases were obtained. The primary upper phase (enriched for right-side out plasma membrane vesicles) was repartitioned twice with fresh lower phases. Likewise, the primary lower phase containing endomembranes and inside-out plasma membrane vesicles was repartitioned twice with fresh upper phases. The final upper and lower phases were diluted in PM washing buffer and the membrane vesicles were pelleted by centrifugation at 180,000g for 30min, and resuspended in 100 and 200µl of PM washing buffer, respectively. Finally, equal volumes of both samples were analysed by Western blotting. As markers for specific membranes we applied following antibodies: H^+^-ATPase for plasma membrane (AS07 260, AGRISERA; www.agrisera.com), V-ATPase for the vacuole (AS07 213, AGRISERA), mitochondrial Porin (kindly provided by Harvey Millar), and Sar1 for the ER (AS08 326, AGRISERA).

## *2D-Gel electrophoresis and Western blotting of Arabidopsis root extracts*

The phenol protein extraction method (Isaacs*on et a*l. 2006) was used to isolate root proteins. 200µl 2D-gel sample containing 100µg protein, 7M urea, 2M thiourea, 2% CHAPS, 0.5% IPG buffer (pH 3–11) NL, and 0.002% bromophenol blue was applied on 11 cm Immobiline DryStrips (pH 3–11) NL from GE HEALTHCARE. Focussing was done in an IPGphor from GE HEALTHCARE according to the manufacturer’s instructions. Subsequently, IPG strips were equilibrated 15min in SDS-PAGE equilibration buffer (75mM Tris-HCl at pH 8.8, 6M urea, 2% SDS, 0.002% bromophenol blue) with 100mg DTT, followed by another equilibration for 15min in SDS-PAGE buffer containing 250mg iodoacetamide. After separation on 8 - 15% gradient SDS-PAGE in the second dimension, proteins were transferred to PVDF membranes by semi-dry blotting. Efficiency of transfer was verified by Ponceau staining and the anti-phosphothreonine antibody (CELL SIGNALLING TECHNOLOGY; www.cellsignal.com) was applied according to the manufacturer’s instructions. For detection of the secondary antibody the SuperSignal® WestPico Chemiluminescent Substrate (PIERCE; www.piercenet.com) was used. After detection membranes were stained with Coomassie brilliant blue R-250.

*Enzymatic digest, LC-MS/MS analysis and data analysis*

Coomassie stained gel spots were excised from the 2D gel and used for nano-electrospray LC-MS/MS investigations after destaining in a mixture of MetOH and 50mM NH_4_HCO_3_. Proteins were reduced by DTT and alkylated by iodoacetamide before tryptic digest overnight at 37 °C. Peptides were separated on a reversed phase column (PepMap C18) with an UltiMate™ HPLC system (DIONEX CORPORATION; www.dionex.com). Linear gradient of ACN and 0.1% formic acid was used at a flow rate of 300nl∙min^-1^. The LC system was directly interfaced to an LTQ (THERMO; www.thermofisher.com) linear ion trap mass spectrometer. Peptide spectra were recorded over the range of m/z 450-1600 with a default charge state of 3. The mass range for MS/MS measurements was calculated according to the masses of the parent ions. Automatic gain control was applied and the collision energy was set to the arbitrary value of 35. Fragmented ions were set onto an exclusion list for 20 seconds. MS/MS spectra were interpreted by Mascot 2.2.04 (MATRIX SCIENCE, www.matrixscience.com) and Bioworks 3.3 (THERMO). Peptide tolerance was set to +/- 2 Da, MS/MS tolerance was set to +/- 0.8 Da and the TAIR9 database was used for search: (<ftp://ftp.arabidopsis.org/home/tair/Sequences/blast_datasets/TAIR9_blastsets/TAIR9_pep_20090619>) Xcorr versus charge state was set as filter for Bioworks with following values: 1.5, 2.00, 2.50 and 3.00 for singly, doubly, triply and four times charged ions respectively. Mascot identifications required at least ion scores greater 20.

As measurement for over-representation of CDPK consensus phosphorylation sites, we analysed the identified proteins for the presence of the following five overlapping CDPK phosphorylation motives: [S]-X-[KRP], [RK]-X-X-[ST]-X-[KRP], [KR]-X-X-[ST], [AL]‑X-[KR]­-­X-X-S-X-[KRP], or [RKHYCDE]-X-X-[KR]-X-X-S-X-X-[KR] according to (Hua*ng et a*l. 2001, Chen*g et a*l. 2002, Hernandez Sebasti*a et* al. 2004). The phosphorrylated residue is underlined.

The overrepresentation of these motifs was compared to the entire *Arabidopsis* proteome by summing up the probability for each motif to appear as compared to the entire proteome and normalising to the protein length. We termed the resulting figure *p*-Score of a protein X of the length l as

(X) **=**

∑

1

5

*a*

*i*

*m*

*i*

100

l

*p-*Score

Were the motif i occurs ai times in a given protein X with the probability mi to occur by chance in the total *Arabidopsis* proteome.
